# Oxidative Stress and Marine Carotenoids: Application by Using Nanoformulations

**DOI:** 10.3390/md18080423

**Published:** 2020-08-13

**Authors:** Yasin Genç, Hilal Bardakci, Çiğdem Yücel, Gökçe Şeker Karatoprak, Esra Küpeli Akkol, Timur Hakan Barak, Eduardo Sobarzo-Sánchez

**Affiliations:** 1 Department of Pharmacognosy, Faculty of Pharmacy, Hacettepe University, Sıhhiye, 06100 Ankara, Turkey; ygncyasin@gmail.com; 2 Department of Pharmacognosy, Faculty of Pharmacy, Acibadem Mehmet Ali Aydınlar University, 34752 Istanbul, Turkey; hilal.bardakci@acibadem.edu.tr (H.B.); Timur.Barak@acibadem.edu.tr (T.H.B.); 3 Department of Pharmaceutical Technology, Faculty of Pharmacy, Erciyes University, 38039 Kayseri, Turkey; cyucel@erciyes.edu.tr; 4 Department of Pharmacognosy, Faculty of Pharmacy, Erciyes University, 38039 Kayseri, Turkey; gskaratoprak@erciyes.edu.tr; 5 Department of Pharmacognosy, Faculty of Pharmacy, Gazi University, Etiler, 06330 Ankara, Turkey; 6 Instituto de Investigación e Innovación en Salud, Facultad de Ciencias de la Salud, Universidad Central de Chile, Santiago 8330507, Chile; 7 Department of Organic Chemistry, Faculty of Pharmacy, University of Santiago de Compostela, 15782 Santiago de Compostela, Spain

**Keywords:** bioavailability, carotenoids, marine, nanoformulation, oxidative stress, reactive oxygen species

## Abstract

Carotenoids are natural fat-soluble pigments synthesized by plants, algae, fungi and microorganisms. They are responsible for the coloration of different photosynthetic organisms. Although they play a role in photosynthesis, they are also present in non-photosynthetic plant tissues, fungi, and bacteria. These metabolites have mainly been used in food, cosmetics, and the pharmaceutical industry. In addition to their utilization as pigmentation, they have significant therapeutically applications, such as improving immune system and preventing neurodegenerative diseases. Primarily, they have attracted attention due to their antioxidant activity. Several statistical investigations indicated an association between the use of carotenoids in diets and a decreased incidence of cancer types, suggesting the antioxidant properties of these compounds as an important factor in the scope of the studies against oxidative stress. Unusual marine environments are associated with a great chemical diversity, resulting in novel bioactive molecules. Thus, marine organisms may represent an important source of novel biologically active substances for the development of therapeutics. Marine carotenoids (astaxanthin, fucoxanthin, β-carotene, lutein but also the rare siphonaxanthin, sioxanthin, and myxol) have recently shown antioxidant properties in reducing oxidative stress markers. Numerous of bioactive compounds such as marine carotenoids have low stability, are poorly absorbed, and own very limited bioavailability. The new technique is nanoencapsulation, which can be used to preserve marine carotenoids and their original properties during processing, storage, improve their physiochemical properties and increase their health-promoting effects. This review aims to describe the role of marine carotenoids, their potential applications and different types of advanced nanoformulations preventing and treating oxidative stress related disorders.

## 1. Introduction

The World Health Organization revealed that approximately 80% of the world’s population count on medicinal plants, in order to maintain their health or for treatment purposes. Medicines and nature have been strictly linked through the utilization of traditional medicines as therapeutic agents for thousands of years. Plenty of studies were performed on traditional medicines, which were primarily plants, constituting the basis of most early medicines (such as aspirin, digitoxin, morphine, quinine, etc.) and providing a pivotal role in today’s drug discovery [1]. Even today, natural metabolites play a crucial role as one of the major sources of novel medicines due to their incomparable structural diversity, relatively small dimensions (<2000 Da), and their drug-like properties (absorption and metabolism) as well [2].

Marine flora and fauna, such as algae, bacteria, sponges, fungi, seaweeds, corals, diatoms, etc. serve as a generous source of bioactive metabolites with a great difference and complexity. The variance of marine environments, sea, and oceans offer a limitless biodiversity in compounds obtained from marine species. In order to survive in extreme habitats, they have developed particular secondary metabolic pathways to produce molecules to accommodate their lifestyles. For example, despite the unusual environmental conditions (light and oxygen exposure) that might lead oxidative damage, marine organisms do not undergo any serious photodynamic damage. Hence, it is known that marine organisms are able to synthesize molecules with bioactivity, especially antioxidant molecules, to protect themselves from external factors, such as ultraviolet (UV) radiation, stress, and herbivores [3,4]. Marine organisms attracted the interest of scientists due to the substantial bioactivities of their extracts and isolates. In this purpose, a number of metabolites of a wide variety of chemical classes, including terpenes, shikimates, polyketides, acetogenins, peptides, alkaloids, and many unidentified and uncharacterized structures, were purified from marine bio resources and exhibited several utilizations as nutraceuticals and pharmaceuticals [2]. Those compounds have various pharmacological activities, such as antioxidant, antibacterial, antitumor, antiviral, anti-inflammatory, antidiabetic, antihypertensive, anticoagulant [4]. Amongst them carotenoids have become the topic of great interest for pharmaceutical industry; thus, they have significant antioxidant activities and anticancer activities [5,6,7,8].

Carotenoids are naturally occurring lipophilic pigments responsible for the red, orange and yellow color of some species of archaea and fungi, algae, plants, and photosynthetic bacteria as well. They are in the structure of tetraterpene and capable of absorbing light primarily between 400 nm and 500 nm. They are encountered in macroalgae, bacteria, and unicellular phytoplanktons and perform diverse and notable functions such as protecting chlorophyll via absorbing light energy and transferring it to chlorophyll and scavenging free radicals of oxygen [9]. Animals are not capable of synthesizing carotenoids de novo since they need to ingest carotenoids via supplementation or in food. Aquatic animals ingest carotenoids from foods, such as algae and other animals, and convert their structure via metabolic reactions leading structural diversity. To date, more than 850 carotenoids were detected in nature, including up to 100 that are present in the food chain and human nutrition, and more than 250 are of marine origin [10,11,12]. The importance of carotenoids is due to their functional properties, not only as natural antioxidants and color enhancing agents in the food industry, but also as pharmaceuticals and as chemotaxonomic markers [8]. Carotenoids play significant role in eye, bone, and cardiovascular health; they are used in cancer prevention, to boost immune function and cognitive performance, as infant nutrition, and antioxidant, antitumour, antiaging, and anti-inflammatory agents. Carotenoids exert their activities via different mechanisms. For example, α-carotene and β-carotene are converted to vitamin A in the human body, and show its activity, lutein, and zeaxanthin protect human eye by absorbing blue and near UV light [8,9,11]. However, technically, the reason behind the other activities of carotenoids is thought to be due to their significant antioxidant activity.

Oxidative stress leads the formation of reactive oxygen species (ROS) against the endogenous and exogenous stimuli. Under physiological conditions, ROS are repeatedly generated and eliminated through ROS scavenging systems in order to maintain redox homeostasis. Change in redox balance leads altered ROS production, resulting in cell damage, aberrant cell signaling; thus, disruption of cell homeostasis [13]. ROS are extremely hazardous for living organisms, causing detrimental diseases, such as cardiovascular diseases, cancer, and diabetes. ROS involve in carcinogenesis through inducing persistent DNA injuries and mutations in p53, the tumor suppressor gene, genomic instability, and aberrant pro-tumorigenic signaling; thus, they might be considered as oncogenic. Thus, prevention of ROS production and balancing antioxidant system is thought to inhibit cancer development. On the contrary, many chemotherapeutic agents in cancer therapy, as well as ionizing radiation, function by promoting ROS production and promoting apoptosis and necrosis of the cells. For instance, some molecules, such as paclitaxel, are able to attack cancer cells via inducing ROS generation or interfering ROS metabolism [14]. Since high levels of ROS are also toxic to cancer cells and potentially induce cell death [13,14]. There are plenty of clinical researches revealing the anticancer and activity of antioxidants; hence, counterbalancing the ROS mediated injury is extremely important in the prevention of plenty of diseases, including cancer [15].

Antioxidants often refer to compounds that are able to donate an electron and neutralize free radicals resulting in scavenging and preventing cell injuries. Carotenoids are not only essential antioxidants, they are also crucial anticancer agents. They exert their anticancer activity via promoting gap junctional communication. Carotenoids initiate cell proliferation and differentiation by binding and regulating the receptors (retinoid acid receptor (RAR) and retinoid X receptor (RXR)). Although they have significant pharmacological activities, there are several limitations of carotenoids, such as low solubility in water, easy degradation, low shelf life, and unfavorable pH in digestive tract leading to alleviated bioavailability. To overcome these undesirable properties, various encapsulation methods are preferred for carotenoids [9]. In this paper, chemical structures, sources, bioavailability, and activities of carotenoids from marine organisms are overviewed.

## 2. Chemical Structures of Carotenoids

**Figure 1:**
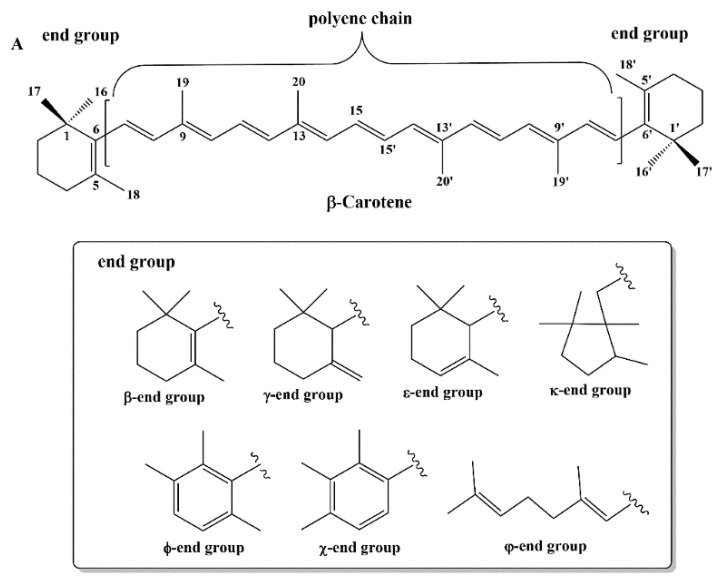
Typical structures of carotenoids.

Most carotenoids are tetraterpenoid compounds consisted of a sequence of eight isoprene units. Biosynthesis of carotenoids include condensation reactions begin with the basic C5 unit dimethyl allyl diphosphate (DMADP) and isopentenyl diphosphate (IDP) units. Prenyl transferases lead the formation of several intermediates that are the origin of the biosynthesis branches for the formation of mono-, di-, and triterpenes. Subsequent tail to tail condensation of two geranylgeranyl diphosphate units to the head to tail condensation of DMADP and IDP to geranyl diphosphate evoke the synthesis of phytoene [16]. Nevertheless, the core structure is a polyene backbone with a series of conjugated C=C double bonds and an end group at both ends of this chain [12]. Typical structures of carotenoids are shown in Figure 1.

**Figure 2:**
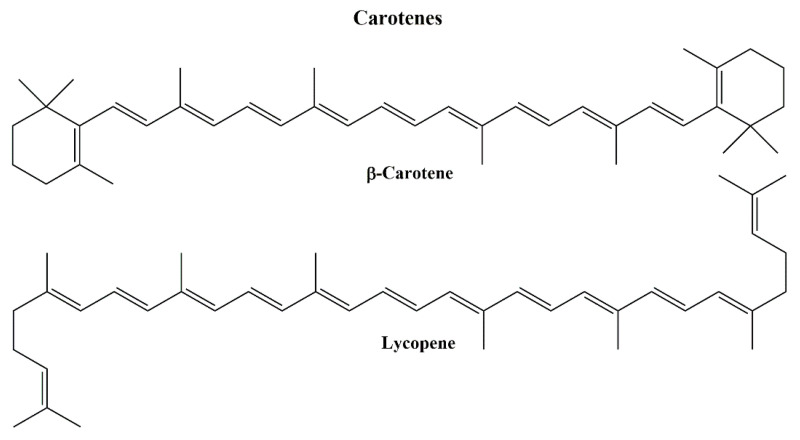
Structures of β-carotene and lycopene.

Currently, carotenoids are classified according to the presence of oxygen; carotenes without oxygen (pure hydrocarbon), and xanthophylls with oxygen in their chemical structure. Parent carotenoid lycopene is produced after stepwise desaturation of phytoene. Lycopene cyclases might interfere the formation of rings at both ends and produce carotenes such as α-carotene, β-carotene, β,ψ-carotene (γ-carotene) (Figure 2). There are about 50 types of carotenes detected in nature [12].

**Figure 3:**
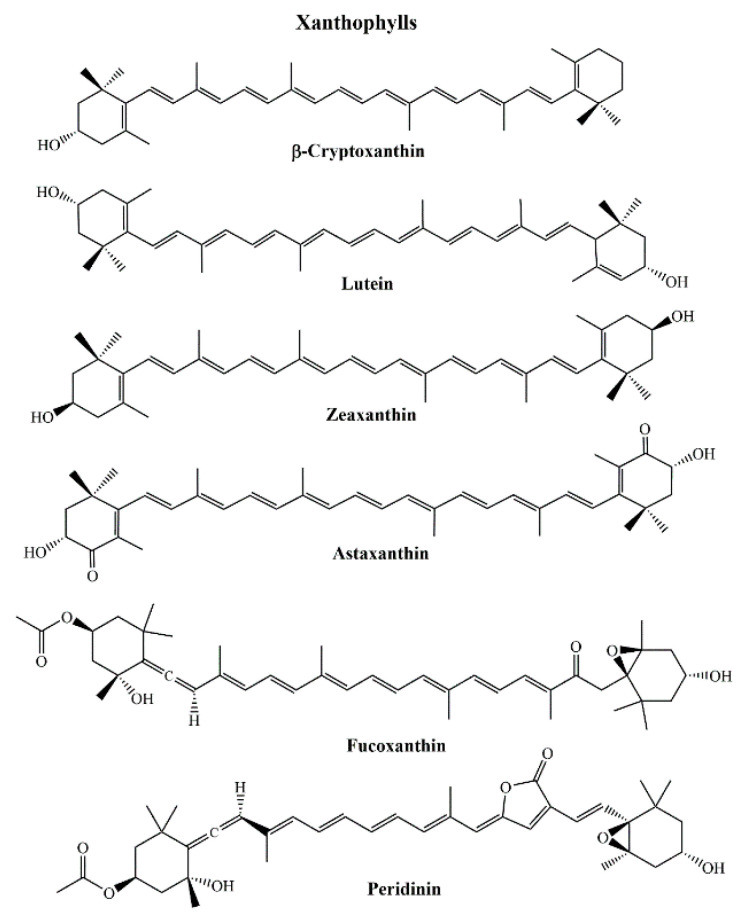
Structures of the examples of common xanthophylls.

Xanthophylls are often characterized by the occurrence of carbonyl, carboxyl, hydroxyl, and epoxide groups in which pairs of hydrogen atoms are replaced with oxygen atoms. More than 800 kinds of xanthophylls have been reported in nature till today. β-cryptoxanthin, lutein, zeaxanthin, astaxanthin, fucoxanthin, and peridinin (Figure 3) are the examples of common xanthophylls. Although there are acyclic (e.g., lycopene) carotenoids are present, more common are with six membered (or rarely five-membered) ring at one end or both ends (bicyclic) of the molecule. [12,16,17]. *Cis*/*trans* isomerization is seen regarding to the stereochemistry of carotenoids. Biosynthetic pathways principally lead the *trans* configured carotenoids to be dominant in nature, although there are few examples of natural *cis* derivatives [16].

**Figure 4:**
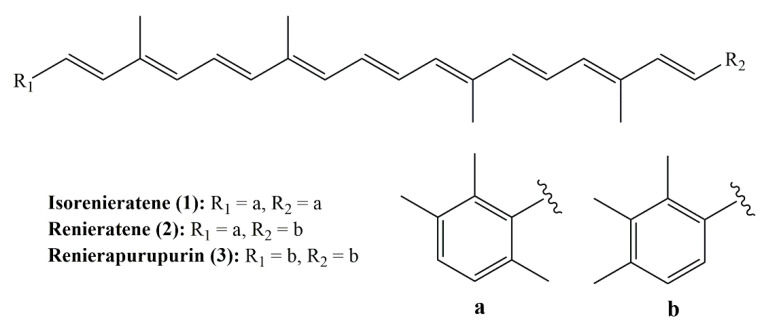
Structures of isorenieratene, renieratene, and renierapurpurin.

Marine sponges are generally in brilliant colors due to the occurrence of carotenoids. They are often associated with symbionts such as bacteria or microalgae. Isorenieratene, renieratene, and renierapurpurin (Figure 4) are the frequently encountered aryl carotenoids in marine sponges.

To date, more than twenty aryl carotenoids have been detected in sponges. Since those compounds also reported in green sulfur bacteria, it was assumed that carotenoids in sponges originated from symbiosis with bacteria [10]. 2-nor-astaxanthin and actinioerythrin are the examples of characteristic carotenoids in sea anemones [10]. Lutein, zeaxanthin, fucoxanthin, and their metabolites are the mainly occurring carotenoids in chitons. β-carotene, α-carotene, zeaxanthin, lutein, and fucoxanthin are primarily encountered on the shells of abalone, and turban shell. Astaxanthin is the principle carotenoid synthesized from β-carotene by many crustaceans’ algae. They ingest β-carotene from dietary algae astaxanthin—it is also widely encountered in both marine and fresh water fish [10].

## 3. Sources of Carotenoids from Marine Organisms

Carotenoids are very common in nature and can be found in highly diverse habitats. Terrestrial vegetables and fruits are important sources of carotenoids. Nonetheless, numerous marine organisms are likewise valuable sources for them. Both prokaryotic and eukaryotic divisions in marine environment may contain sufficient amount of various carotenoids. Prokaryotes are considered as primitive living organism in phylogenetic classification. However, there are several carotenoid containing members of this division. For example, Agrobacterium and Paracoccus genera in bacteria kingdom are favorable sources of astaxanthin [18]. In another study, two rare carotenoids with promising biological activities, saproxanthin and myxol were isolated from marine bacteria belonging to Flavobacteriaceae family. Members from Archaea division may also be valuable sources of carotenoids. Several studies demonstrated that, halophilic archaea from Haloferacaceae family can produce β-carotene, phytoene, lycopene, bacterioruberin, and salinixanthin [19]. *Cyanobacteria phylum*, photosynthetic microorganisms of prokaryotic division are widespread in various marine environments are also important carotenoid producers. Zeaxanthin, synechoxanthin, canthaxanthin, echinenone, nostoxanthin, caloxanthin, and myxoxanthophyll are some examples of carotenoids which were found in *Cyanobacteria phylum* [20].

Carotenoids are present in every kingdom of eukaryotes division: Protista, Fungi, Plantae and Animalia. Thraustochytrids family in Protista kingdom has carotenoid producers. Several strains from genera Thraustochytrium, Ulkenia, and Aurantiochytrium synthetize various carotenoids such as β-carotene, astaxanthin, zeaxanthin, canthaxanthin, phoenicoxanthin, and echinenone [21]. Furthermore, various yeast species were found from marine habitats that produce carotenoids especially Astaxanthin. Xanthophyllomyces, Rhodotorula, and Phaffi genera can be used for Astaxanthin production; however, amounts are lower when compared to algae [22].

Microalgae and macroalgae are considered main source of marine carotenoids. Their carotenoids accumulation can be due to their essential metabolism for survival (primary carotenoids) or precise ecological pressure (secondary carotenoids) [23]. The Chlorophyceae family contains most important genera for carotenoid production. *Dunaliella salina* from Chlorophyceae family is considered as a source of β-carotene production when it encounters with extreme conditions, such as high light density, nutrition deficiency, and salinity. It can also produce α-carotene, lutein, and zeaxanthin but is generally used for large scale β-carotene production [24]. Genus *Chlorella* and *Scenedesmus* genera are prominent sources of lutein therefore they are used in large scale of lutein production [25]. *Tetraselmis suecica* is marine green algae, which is generally used for aquaculture nutrition and rich for tocopherol [26]. *Haematococcus pluvialis* is widely used for astaxanthin production for a long period of time [27]. Chlamydomonas and Muriellopsis are some other examples of carotenoid producing microalgae and also lycopene, fucoxanthin, canthaxanthin, echinenone, and dinoxanthin are some examples of carotenoids from the Chlorophyceae family [28]. Macroalgae (seaweeds) are also noticeable sources of carotenoids, such as fucoxanthin, lutein, β-carotene and siphonaxanthin [29]. Especially brown algae species are important sources of fucoxanthin. *Hijikia fusiformis*, *Sargassum* sp., *Undaria pinnatifida*, *Fucus* sp., *Laminaria* sp., *Alaria crassifolia*, *Ishige okamurae*, *Cystoseira hakodatensis*, *Eisenia bicyclis*, *Myagropsis myagroides*, *Cladosiphon okamuranus*, *Petalonia binghamiae*, *Hijikia fusiformis*, *Kjellmaniella crassifolia,* and *Padina tetrastromatica* are important examples for their fucoxanthin content [30]. Seagrasses are also important carotenoid sources. Previous articles demonstrated that *Posidonia oceanica*, *Cymodocea nodosa*, *Zostera noltii*, *Halophila stipulacea*, *Cymodocea nodosa,* and *Zostera marina* are producers of several carotenoids such as β-carotene, lutein, lutein epoxide, zeaxanthin, violaxanthin, neoxanthin, siphonaxanthintype, and violaxanthin [31,32].

Even though marine animals do not synthetize carotenoids de novo, they can accumulate carotenoids via direct food intake or small metabolic transformations. Carotenoids are generally found in invertebrate marine animals in phylums Pomifera, Cnidaria, Mollusca, Crustacea, Echinodermata, and Tunicata. Sponges (Pomifera) contain more than 40 carotenoids, generally aryl carotenoids such as renierapurpurin, renieratene and isorenieratene which are assumed that occurs after biotransformation of fucoxanthin [33]. Phylum Cnidaria has small number of members contains carotenoids. *Actinia equine*, *Tealia feline*, and *Anemonia sulcata* are some examples that accumulate rare carotenoids such as 2-nor-astaxnthin, peridinin and actinioerythrin [34]. Mollusks contain numerous types of carotenoids. It has been shown that shellfishes contain β-carotene, lutein A, zeaxanthin, diatoxanthin, astaxanthin, etc. [34]. Bivalves and chitons also contain several carotenoids including fucoxanthin, zeaxanthin, lutein, diaxanthin, and peridinin [35]. In Crustacea phylum, crabs, shrimps, and lobsters generally accumulate astaxanthin, which is biotransformed from β-carotene after consumption of algae [35]. Sea urchins, holothurians, and starfishes from Echinodermata contain several carotenoids such as β-carotene, echinenone, canthaxanthin, astaxanthin and fucoxanthin [30]. Tunicates also biotransform carotenoid obtained from phytoplankton consumption, such as alloxanthin, fucoxanthin, mytiloxanthin, mytiloxanthinone, and halocynthiaxanthin [36]. Moreover, salmon fish accumulates astaxanthin in their muscles, which makes them valuable dietary carotenoid source [35].

## 4. Oxidative Stress and Cancer

Reactive oxygen species formed intracellular (mitochondria, the endoplasmic reticulum (ER), peroxisomes, microsomes, phagocytic cells, NAPDH oxidase (NOX) complexes, etc.), or extracellular sources. Specifically, mitochondria, produce significant amounts of reactive oxygen species (ROS), which can contribute to intracellular oxidative stress [37]. Another important source is NADPH oxidases found in various cells, especially phagocytes and endothelial cells, which are central to the formation of the inflammatory response [38]. Extracellular sources of ROS generation include ROS-inducing agents, are generally UV radiation, chemical compounds like drugs and toxins, pollutants, cigarette and alcohol [39]. The examples for the radicals include Superoxide (O_2_^⋅−^), Oxygen radical (O_2_^∙∙^), Hydroxyl (OH^∙^), Alkoxy radical (RO^∙^), Peroxyl radical (ROO^.^). The high reactivity of these radicals is due to the presence of an unpaired electron that tends to donate it or tends to obtain another electron to achieve stability. The non-radical species include hydrogen peroxide (H_2_O_2_), hypochlorous acid (HOCl), hypobromous acid (HOBr), ozone (O_3_), singlet oxygen (^1^O_2_). Molecular oxygen (O_2_) has two unpaired electrons with a parallel spin state and tends to form highly ROS. While molecular oxygen reacts slowly with non-radical substances, it reacts easily with other free radicals [40].

Molecular oxygen (O_2_) has two unpaired electrons with a parallel spin state and tends to form highly ROS. While molecular oxygen reacts slowly with non-radical substances, it reacts easily with other free radicals. Reactive nitrogen types, like reactive oxygen species, occur in the biological environment when free radicals form more stable species with many effects [41].

These radicals damage DNA and proteins, causing cancer and oxidizing LDL, leading to cardiovascular diseases. Free radicals are not completely harmful in fact; our body also needs free radicals in the production and activation of some vital hormones [42]. Free radicals also come into play in the body in case of infection and removal of foreign substances, but they can also cause damage to the structure of healthy cells, such as DNA protein, and lipids and cause many diseases [43]. Bio membranes and intracellular organelles are sensitive to oxidative attacks due to the presence of unsaturated fatty acids in membrane phospholipids. When lipid peroxides formed by oxidation of lipids are destroyed, aldehydes, most of which are biologically active, are formed. These compounds are either metabolized at the cell level or diffuse from their original domains and spread damage to other parts of the cell. Malondialdehyde (MDA) occurs in the peroxidation of fatty acids containing three or more double bonds. MDA and 4-hydroxy-2-nonenal (4-HNE) formed as a result of lipid peroxidation are known to react with nucleotides and cause mutagenesis and carcinogenesis [44,45]. Amino acids that make up proteins tend to oxidize to a higher degree than lipids in general because they contain carboxyl and amino groups. These molecules contain reduced carbon atoms that will undergo oxidative transformation in their side chains. In many studies, diseases such as Parkinson’s, diabetes, Alzheimer’s, renal tumor formation and rheumatoid arthritis have been associated with increased protein carbonyl groups [46].

**Scheme 1:**
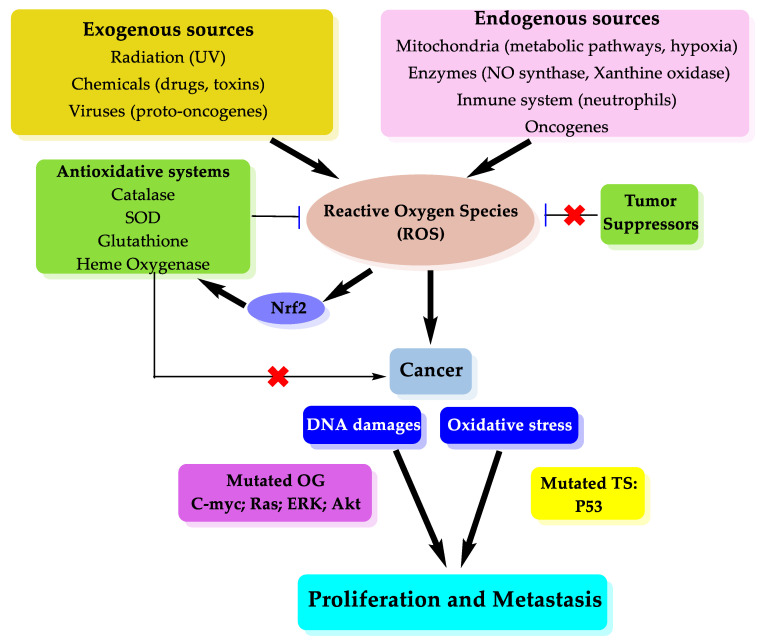
Oxidative stress and cancer metastasis relation.

The main target of oxidative damage in the DNA chain is purine, pyrimidine bases and sugar structure [47]. Moreover, 8-hydroxy-2-deoxyguanosine is generated via reactive species and this product can produce mutations in DNA, which has carcinogenesis effect [48]. Disequilibrium between the production and elimination of ROS is defined as oxidative stress and numerous studies have shown the important roles of oxidative stress in propagation of cancer [49]. ROS can trigger many aspects of tumor development and progression, which can be divided into the cellular proliferation, evasion of apoptosis, tissue invasion and metastasis and angiogenesis. ROS can trigger number of signaling pathways like extracellular-regulated kinase 1/2 (ERK1/2), receptor tyrosine kinase (RTK), Wnt, Src, NF-κB, phosphatidylinositol-3 kinase (PI3K/Akt), matrix metalloproteinase (MMP), hypoxia inducible factor-1α (HIF-1α), and vascular endothelial growth factor (VEGF) [45,48]. Permanent oxidative stress due to high ROS level in antioxidant system weakness can activate Akt, ERK and c-MYC like oncogenes by inhibiting tumor suppressors such as p53 and Phosphatase and tensin homolog deleted on chromosome 10 (PTEN) [45]. ROS also play an important role in the spread of cancer and tumor formation in a secondary place. In the case of metastasis, ROS can interact with the cytoskeleton and extracellular matrix. In case of oxidative stress MMPs activated and they inhibit movement that suppresses metastasis (Scheme 1) [50].

## 5. Carotenoids and Oxidative Stress

The capacity of carotenoids to quench O_2_ and scavenge free radicals has been proposed as the main mechanism by which they afford their benign health effects [51]. Due to their triplet energy levels lying close to that of ^1^O_2_ (1274 nm, 7849 cm^−1^ or 93.9 kJ/mole vs. 1380 nm, 7250 cm^−1^ or 86.7 kJ/mol for β-carotene, respectively, they are included in the group of the most effective physical quenchers of ^1^O_2_ [52]. Carotenes with 11 conjugated double bonds have especially been shown to possess very strong extinguishing ability of ^1^O_2_ quenching. In general terms, ^1^O_2_ neutralization is based on transforming excess energy into heat through the lowest excited triple state (^3^Crt*) of carotenoids. The possible harmful effects of stimulated carotenoids can often be neglected due to their low energy and short life [53].

^1^O_2_ + Carotenoid→ ^3^O_2_ + ^3^Crt*(1)

^3^Crt* → Carotenoid + heat

Radical scavenging mechanism of carotenoids are: (i) electron transfer, resulting with carotenoid radical cation (Carotenoid ^•+^) or carotenoid radical anion (Carotenoid ^•−^); (ii) radical addition/adduct formation, (R Carotenoid ^•^); and (iii) allylic hydrogen removal with formation of neutral carotenoid radical (Carotenoid ^•^) [52,54].

(i) R^•^ + Carotenoid → R^−^ + Carotenoid ^•+^

R^•^ + Carotenoid → R^+^ + Carotenoid ^•−^

(ii) R^•^ + Carotenoid → R Carotenoid ^•^

(iii) R^•^ + Carotenoid → RH + Carotenoid ^•^

However, some studies have shown that carotenes and xanthophylls have pro-oxidant properties under some conditions. When lipid peroxidation is in progression under high oxygen pressure [55], higher amount of carotene-peroxyl radical (Car-OO^•^) created, and as long as it is not eliminated by different antioxidant systems, Car-OO• will replicate lipid peroxidation with more attack on intact unsaturated fatty acid chains [54].

Because limited absorption and tissue specific accumulation of carotenes, it is wrong to assume that carotenoids will support health by scavenging ROS or RNS. Common belief that the removal of free radicals by antioxidants has a beneficial effect; however, the major contribution of carotenoids and their metabolites to cell protection is presumed to be stimulating endogenous antioxidant defenses like nuclear factor E_2_-related factor 2 (Nrf2−Keap1) pathway [56,57,58]. Lycopene, phytoene, phytofluene, β-carotene and astaxanthin were evaluated for their effect on activation of antioxidant responsive elements (ARE) and their role in the induction of phase II enzymes. It was reported that the efficacy of carotenoids in ARE activation was not related to their effects on intracellular ROS and decreased glutathione level. The increase in Phase II enzymes like NAD(P)H:quinone oxidoreductase (NQO1) and γ-glutamylcysteine synthetase (GCS) has been removed by a dominant negative Nrf2, suggesting that carotenoid induction of these proteins is due to a functional Nrf2 and ARE transcription system [59].

Studies on the radical scavenging and antioxidant activities of various marine carotenoids have been summarized to understand the effects on oxidative stress. Astaxanthin, the xanthophyll carotenoid found in marine organisms, is one of the natural compounds with strong antioxidant activity. Astaxanthin, lycopene, β-carotene, and lutein carotenoids have been studied for their antioxidant activity with fluorometric assay. The 8-tetramethylchroman-2-carboxylic acid (Trolox) was employed as a calibrator in the assay. The results of the research highlighted that astaxanthin possess the highest antioxidant activity against peroxyl radicals [60].

Dose and colleagues studied the radical scavenging and antioxidant activity of synthetic astaxanthin with various methods. According to research, radical scavenging activities specified with 2,2-diphenyl-1-picrylhydrazyl (DPPH) radical scavenging, galvinoxyl radical scavenging, superoxide radical scavenging, and photon counting for singlet oxygen scavenging assays. In human hepatic cellular carcinoma (Huh7) cells, paraoxonase activity and Nrf2 transactivation and in HepG2 cells (hepatocellular carcinoma) glutathione assay and lipid peroxidation via BODIPY assay performed for cellular antioxidant activity. It has been reported that astaxanthin scavenge DPPH, galvinoxyl and singlet oxygen radicals in a concentration dependent manner but no effect on superoxide anion radicals. In cellular antioxidant activity astaxanthin increased the glutathione levels and also decreased lipid peroxidation in HepG2 cells [61].

A recent study also demonstrated the radical scavenging potential of astaxanthin with DPPH, 2,2′-azino-bis(3-ethylbenzothiazoline-6-sulfonic acid) (ABTS) radical scavenging activities and singlet oxygen quenching assays and also antioxidant potential with β-carotene bleaching activity. It was found that EC_50_ value in DPPH assay was 7.5 μg/mL and 7.7 μg/mL for ABTS assay. The research concluded that shrimp astaxanthin is a potent antioxidant agent and it has no cytotoxic effect on human dermal fibroblast cells [62].

Astaxanthin, zeaxanthin, lutein, ascorbic acid, and tocopherol acetate evaluated for their antioxidant activity with spectrophotometric, fluorimetric and chemiluminescence techniques. In dose dependent manner astaxanthin, lutein, and zeaxanthin showed near same activity against H_2_O_2_ and O_2_^-^. In this study, the strong antioxidant activity of xanthophylls was confirmed and it was also reported that they may be the first option to prevent the retinal oxidative stress due to their ability to pass the retina-blood barrier and their binding to the photoreceptor membranes [63]. In 2007, Santocono et al. conducted a new study to measure the protective effects of astaxanthin, lutein and zeaxanthin against K-N-SH human neuroblastoma cells against DNA damage caused by divergent reactive nitrogen species. Cells were treated with 20 and 40 µM of carotenoids. According to the comet assay, data revealed the ability of preventing DNA damage of carotenoids and it is also stated that this activity subjected to the type of RNOS donor and concentration [64].

Antioxidant activity of astaxanthin in the LS-180 cell line (human colorectal cancer). Cells were treated 50,100 and 150 μm astaxanthin. According to the results of the study, astaxanthin induced apoptosis, as well as decreased MDA levels and caused an increase in antioxidant activity with the effect of superoxide dismutase (SOD), catalase (CAT), and glutathione peroxidase (GPx). In the antioxidant activity tests, 150 μM astaxanthin showed significant effect among the other groups [65].

In UVA exposed human dermal fibroblasts, the effects of astaxanthin, canthaxanthin, and β-carotene on ROS and TBARS were evaluated. While radical scavenging activities of β-carotene and canthaxanthin are not observed, astaxanthin has been stated to have significant radical scavenging activity at both 5 and 10 µM doses. The increase in TBARS levels with UVA exposure decreased significantly (70%) compared to the control group with astaxanthin administration. In addition, the reduction of CAT and SOD enzymes in cells after UVA exposure was prevented by astaxanthin and β-carotene, while canthaxanthin had no effect [66].

Antioxidant effect of astaxanthin was evaluated against cyclophosphamide-induced oxidative stress and DNA damage. Rats were administered with 25 mg/kg (p.o) astaxanthin before or after administration of cyclophosphamide. In the control group, due to cyclophosphamide, an increase in MDA level and a decrease in Glutathione (GSH) amount were observed, whereas in pre- and post- treatment group, this situation resolved through astaxanthin [67].

**Figure 5:**
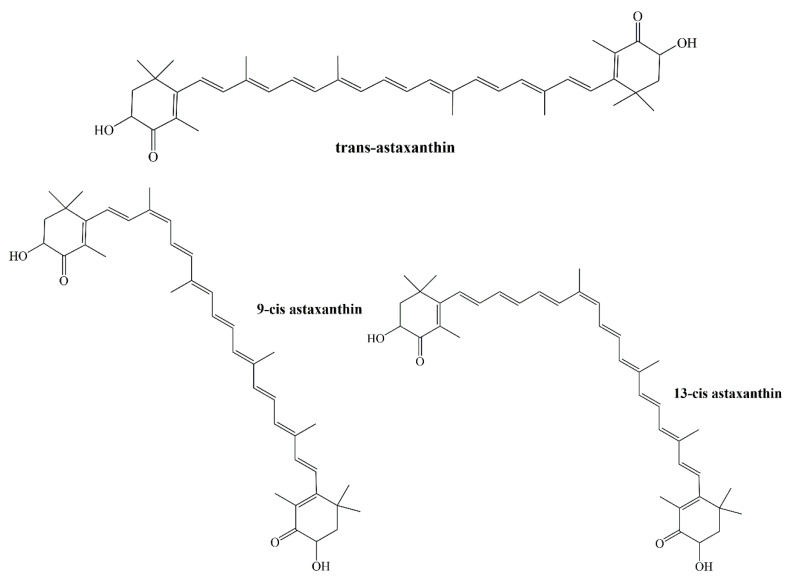
Structures of all-*trans* astaxanthin, 9-*cis* astaxanthin, and 13-*cis* astaxanthin.

DPPH radical scavenging capacity, microsomal lipid peroxidation inhibitory activity, and ROS scavenging activity in SH-SY5Y (human neuroblastoma) cells were performed in a study in which the antioxidant activities of 9-*cis* and 13-*cis* astaxanthin against all-*trans* isomer were measured (Figure 5). 9-*cis* astaxanthin and 13-*cis* astaxanthin scavenge the DPPH radical stronger than the *trans* isomer. In 2,2’-Azobis(2-amidinopropane) dihydrochloride (AAPH) and tert-butyl hydro peroxide (tBuOOH) induced lipid peroxidation, particularly 9-*cis* astaxanthin performed better antioxidant activity by inhibiting TBARS generation than all-*trans* isomer. In SH-SY5Y cells, 100 nM astaxanthin isomers were applied to the cells before 6-hydroxydopamine (6-OHDA) inducement. ROS generation was effectively suppressed by 9-*cis* astaxanthin [68].

Fucoxanthin is a carotenoid belonging to the class of xanthophylls, is common in brown seaweeds, and its effects on oxidative stress have been extensively studied. Iwasaki et al. (2012), conducted a study with purified fucoxanthin from seaweed *Undaria pinnatifida* to highlight the in vivo antioxidant activity. ICR mice and obese/diabetes KK-Ay mice were administered fucoxanthin (0.1%) with soybean oil. Lipid hydro peroxide levels in liver and abdominal white adipose tissue were measured. The 0.1% Fucoxanthin decreased the hydro peroxide amounts in KK-Ay mice. Little change in lipid hydro peroxide levels was observed in ICR mice with and without fucoxanthin administration. The activity of fucoxanthin on lowering lipid hydro peroxides in KK-Ay mice has been attributed to its decreasing effect on blood glucose level and hepatic lipid levels, not its radical scavenging feature [69].

Fucoxanthin purified from *Fucus vesiculosus*, *Fucus serratus* and *Laminaria digitata* brown algae and evaluated for its antioxidant activity. DPPH radical scavenging, iron (Fe^2+^)-chelating activity and reducing power activity were determined. Fucoxanthin had lower DPPH scavenging activity than BHT, while showed similar activity with EDTA in iron chelating activity. In reducing power assay fucoxanthin exhibited significantly lower (*p* < 0.001) activity than ascorbic acid at the same concentration. In the 5% fish oil-water emulsion containing iron as oxidation inducer, fucoxanthin showed better antioxidant activity compared with BHT with low levels of volatile oxidation products and reduction in tocopherol loss [70].

Antioxidant activity of fucoxanthin from *Phaeodactylum tricornutum* microalga examined by DPPH, hydrogen peroxide and superoxide anion radical scavenging activities and also reducing power was evaluated. IC_50_ value of DPPH scavenging activity was found to be 0.30 mM and it has been stated that fucoxanthin was more active than ascorbic acid, BHA, and α-tocopherol positive controls. When compared to positive controls fucoxanthin had lower effect in scavenging the hydrogen peroxide and superoxide anion radicals. In reducing power assay fucoxanthin showed higher activity than positive controls [71]. In a similar study, DPPH radical scavenging IC_50_ value of fucoxanthin was found 201.2 ± 21.4 µg/mL while β-carotene exhibited lower activity. In ferric reducing activity, fucoxanthin and astaxanthin were found to be more active than β-carotene. Results showed that fucoxanthin caused a 63% reduction in chemiluminescence in blood neutrophils and a 3.3-fold increase in reduced and oxidized glutathione in HeLa cells in a dose-dependent manner [72]. Xia et al. reported that fucoxanthin from *Odontella aurita* marine diatom showed potent antioxidant effects in DPPH and ABTS radical scavenging activities. EC_50_ values were found to be 140 and 30 µg/mL, respectively [73]. In a different study conducted by Sujatha et al. (2017), DPPH radical scavenging effect of fucoxanthin from brown seaweed *Sargassum wightii* with an EC_50_ value of 322.58 µg/mL was detected [74]. Fucoxanthin from *Sargassum filipendula* brown seaweed exhibited a strong DPPH radical scavenging activity with 1.4174 ± 0.0126 µg/L EC_50_ value [75].

In a study of Sudhakar et al. (2013), fucoxanthin purified from *Sargassum wightii*, *Sargassum ilicifolium*, *Sargassum longifolium*, *Padina gymnospora,* and *Turbinaria ornata* brown seaweeds. It was mentioned that fucoxanthin purified from *Padina gymnospora* exhibited better activity with 37% inhibition percentage than fucoxanthin isolated from *Sargassum ilicifolium* [76].

Antioxidant activity of fucoxanthin has been studied under anoxic and aerobic conditions via DPPH radical. In the same study also β-carotene, β- cryptoxanthin, zeaxanthin, lycopene and lutein evaluated for their antioxidant activity under anoxic conditions. It was concluded that other carotenoids except fucoxanthin reacted with DPPH at a low level. Under aerobic conditions to observe the stoichiometry of the reactions, 20–300 µM fucoxanthin was added to the 100 µM DPPH. Unlike anoxic reactions, fucoxanthin was found to react less with DPPH [77].

In high fat diet rats, antioxidant activity of fucoxanthin evaluated by Ha et al. (2013). Rats were administered 0.2% fucoxanthin with high fat during 4 weeks. Lipid peroxidation, plasma total antioxidant capacity (TAC), and activities of CAT, SOD, and GPx enzymes were identified. In plasma, total antioxidant capacity and GPx amounts were found to be significantly augmented in fucoxanthin treated group. In liver, CAT and GPx levels in fucoxanthin treated group were also found to be higher than control group. The effect of fucoxanthin on plasma lipid peroxidation did not differ statistically [78].

**Figure 6:**
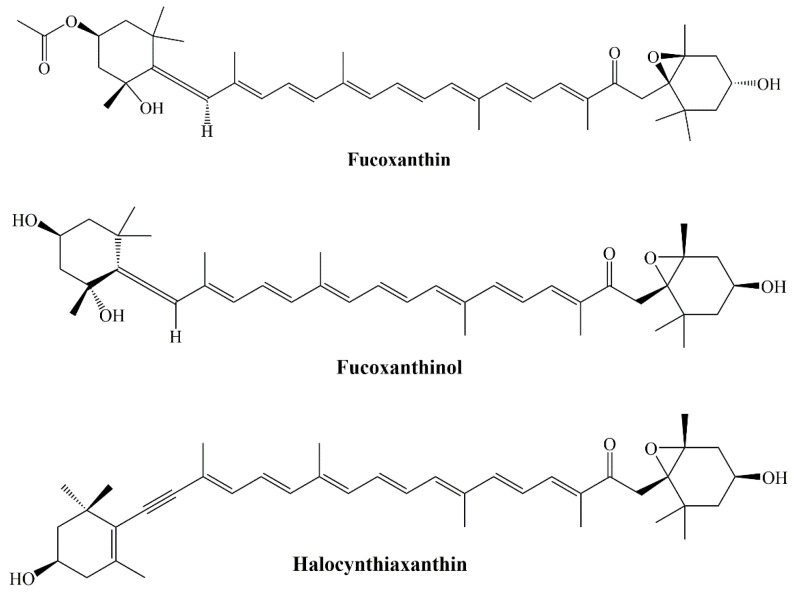
Structures of fucoxanthin and its metabolites.

Fucoxanthin and, fucoxanthinol and halocynthiaxanthin metabolites were analyzed for their DPPH and ABTS radical scavenging activities and singlet oxygen quenching abilities (Figure 6). While fucoxanthin and fucoxanthinol scavenge the DPPH radical stronger than halocynthiaxanthin, fucoxanthinol had a stronger effect against the ABTS radical. In hydroxyl and superoxide radical scavenging activities fucoxanthin exhibited potent scavenging effect than both its metabolites. Moreover, β-carotene was found to be more effective in singlet oxygen quenching than fucoxanthin and its metabolites [79].

In mice with traumatic brain injury (TBI), the effects of fucoxanthin on oxidative stress were investigated by measuring MDA and GPx levels. Mice were administered intragastrically 50, 100, and 200 mg/kg fucoxanthin. Treatment of fucoxanthin, caused a decline in MDA levels and increase in GPx levels in the cerebral cortex tissue of TBI mice. Moreover, in the same study, fucoxanthin has been reported to increase in vitro neuron survival and decrease ROS level [80].

**Figure 7:**
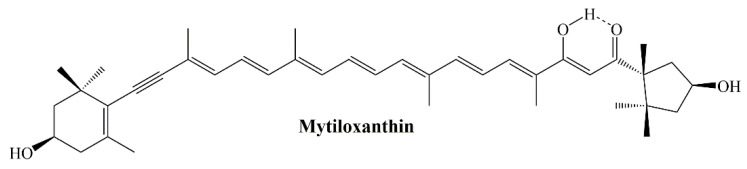
Structure of mytiloxanthin.

Maoka and colleagues synthesized mytiloxanthin, metabolite of fucoxanthin in shellfish and tunicates and evaluated its antioxidant activity by examining the quenching ability of scavenging singlet oxygen, hydroxyl radical and lipid peroxidation inhibition (Figure 7). In the same study, β-carotene, astaxanthin, and fucoxanthin were used as positive controls. Mytiloxanthin exhibited near same singlet oxygen quenching ability (61.6%) with astaxanthin (61.0%). Lower capacity of fucoxanthin in the same study was linked to the number of conjugated double bonds, polyene chain structures, and functional groups. Mytiloxanthin increased its activity by having an 11-conjugated double bond polyene system containing an acetylenic and a carbonyl group in its structure. In the lipid peroxidation experiment, mytiloxanthin exhibited slightly stronger activity than astaxanthin, while fucoxanthin and β-carotene showed higher activity [81].

In the high fat diet rodent model, lutein and zeaxanthin isomers were investigated for their effects on lipid metabolism and anti-inflammatory activities on the retina. Lutein and zeaxanthin used in the experiment provided from Lutemax 2020™. Rats were administered lutein and zeaxanthin 100 mg/kg BW for 8 weeks. Lutein and zeaxanthin improved oxidative damage by decreasing the concentration of MDA and gaining the SOD, CAT, and GPx enzyme activities of the retina caused by high fat diet [82].

Zou et al., conducted a study for evaluating the regulation mechanism of zeaxanthin on phase II detoxification enzymes in human retinal pigment epithelium cells. It has been found that zeaxanthin is effective against mitochondrial dysfunction and apoptosis due to tert-butyl hydroperoxide, while it increases GSH by Nrf2 activation. In the same study, the effects of zeaxanthin were evaluated in the rat retina, liver and heart in vivo. Similar to in vitro results zeaxanthin was found to be effective in increasing GSH levels and decreasing markers of lipid and protein peroxidation, 4-hydroxynonenal and the carbonyl protein [83].

**Figure 8:**
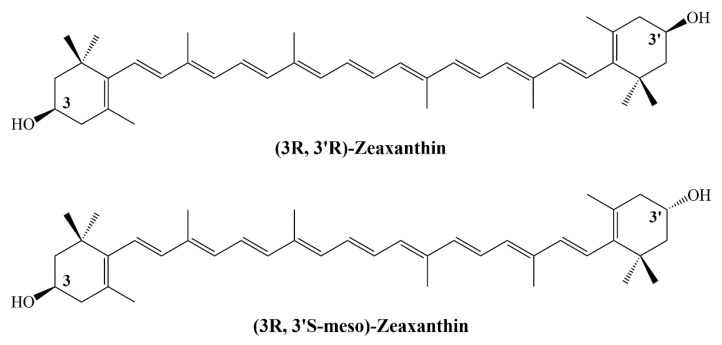
Structure of zeaxanthin.

Bhosale et al., studied the antioxidant role of zeaxanthin and glutathione S-transferase (GSTP1) in egg yolk phosphatidylcholine liposomes. The two zeaxanthin diastereomers showed synergistic antioxidant effects against both azo lipid peroxyl radical producers [(2,2′-azobis(2-methyl-propionamidine) dihydrochloride and lipophilic 2,2′-azobis(2,4-dimethylvaleronitrile)] when bound to GSTP1. Non-dietary (3*R*,3′*S*-meso)-zeaxanthin was found to have strong activity than dietary (3*R*,3′*R*)-zeaxanthin in the presence of GSTP1 (Figure 8) [84].

Singlet oxygen quenching abilities of lycopene, β-carotene, zeaxanthin, astaxanthin, and canthaxanthin carotenoids evaluated by Cantrell et al. (2003). Lycopene showed the fastest quenching ability with 2.3 × 109 M^−1^ s^−1^ value, while the lowest activity is attributed to lutein 1.1 × 108 M^−1^ s^−1^ value. Interestingly, the ability of zeaxanthin to extinguish singlet oxygen decreases as concentration increases [85].

Several studies stated the beneficial effects of dietary supplementation of canthaxanthin the breeder’s diet. Results clearly showed that increased antioxidative status in the egg yolk and newly hatched chicks and as a result hatching rate of chicken eggs was meaningfully increased. The common results of the studies focused on canthaxanthin providing great benefits for chicken eggs, embryos and chickens during postpartum development [86,87,88,89,90].

Canthaxanthin isomers which are purified from new soil *Dietzia* sp. evaluated for their antioxidant activity. DPPH and superoxide radical scavenging activities in addition ROS inhibitory activity in THP-1 (monocytic cell line) cells were performed. It was reported that the *cis* isomer of canthaxanthin showed 1.8-fold higher activity than *trans* isomer in DPPH radical scavenging activity. The *cis* isomer was found to be more active in superoxide radical scavenging activity and inhibiting the ROS formation in THP-1 cells as well [91].

In the study investigating the antioxidant activity of β-carotene used orally in patients with cystic fibrosis, groups were established with 24 patients and 14 healthy individuals. The total antioxidant capacity in the plasma of the 13 cystic fibrosis supplement group increased by 12% after 12 weeks of supplementation. Antioxidant activity has been demonstrated by a reduction in the amount of plasma MDA [92].

Allard et al. (1994) previously conducted a similar study on smoker and non-smoker volunteers. Lipid peroxidation was quantified by breath-pentane output and it was found that lipid peroxidation was significantly lower in smokers who received β-carotene compared to the control group [93]. However, it has been reported in several studies that high amounts of β-carotene supplementation cause adverse effects in people which exposed to high levels of oxidative stress [94].

**Figure 9:**
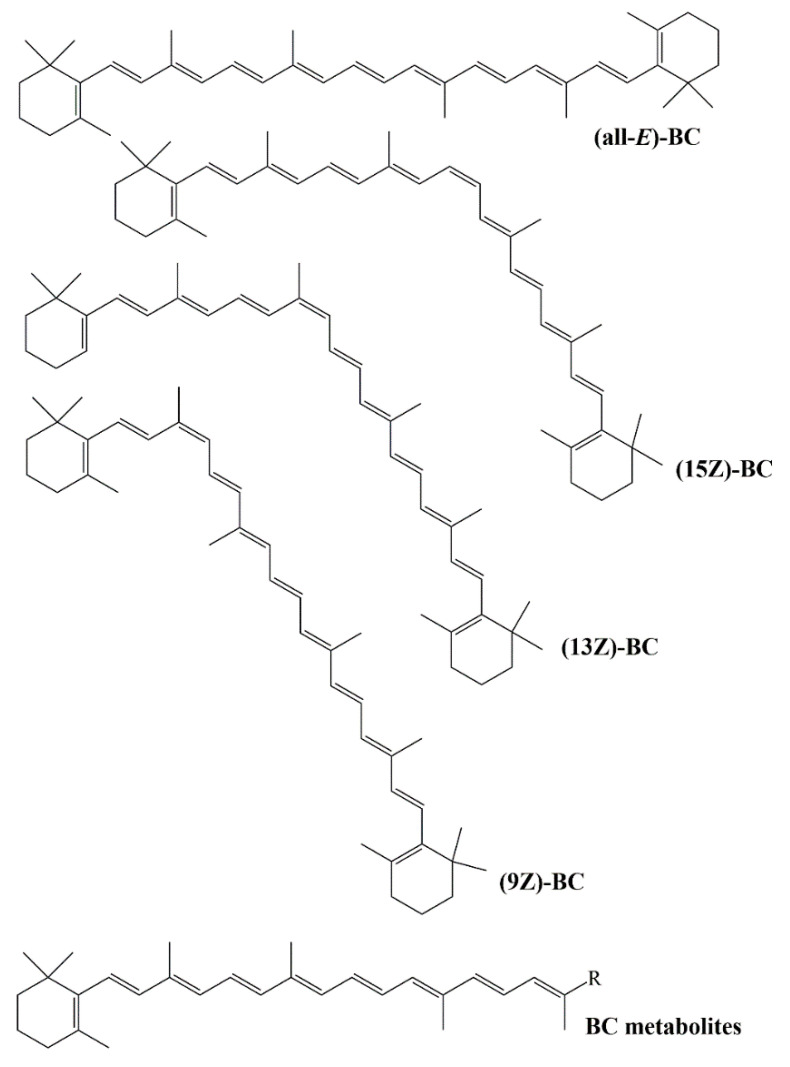
Structures of β-carotene isomers.

Levin et al. (1997) studied with 9-*cis* β-carotene and all-*trans* β-carotene to compare their antioxidant activities. Isolated 9-*cis* β-carotene from *Dunaliella* and synthetic all-*trans* β-carotene administered to rats 1 g/kg. It has been reported that *cis* isomer has a greater affinity for free radicals in the liver. Levin et al. also reported the higher antioxidant activity of 9-*cis* β-carotene than all-trans β-carotene by avoiding methyl-linoleate peroxidation and algal carotene degradation in vitro. However, in some studies, it has been reported in contrasting results regarding the antioxidant activities of β-carotene and isomers (Figure 9) [95]. In the study of Mueller et al., it was explained that β-carotene isomers do not have ferric reducing activity. In addition, it has been reported that the ABTS and superoxide radical scavenging activities of all-*E*, 9*Z* and 13*Z* isomers did not differ statistically, while the 15*Z* isomer had lower activity [96].

Similarly, in the study conducted by Rodrigues et al. (2012), it was reported that *cis*-β-carotene isomers have lower peroxyl radical scavenging activity than *trans*-isomers, and *trans* lycopene is the most active carotenoid among the studied carotenoids [97].

**Figure 10:**
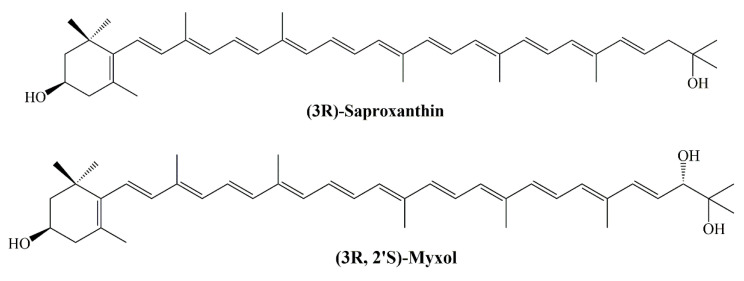
Structures of (3*R*)-saproxanthin and (3*R*,2′*S*)-myxol.

The rare marine carotenoids (3*R*)-saproxanthin and (3*R*,2′*S*)-myxol (Figure 10) have been isolated from 04OKA-13-27 (MBIC08261) and YM6-073 (MBIC06409) two marine bacteria strains and these strains have been classified in the Flavobacteriaceae family. Their inhibitory activity on lipid peroxidation in rat brain homogenate was evaluated and zeaxanthin was also studied in the same experiment. According to the results, IC_50_ values of saproxanthin, myxol, and zeaxanthin were found to be 2.1, 6.2, and 13.5 μM, respectively [98].

Conjugated double bond system forms most of the basic properties of carotenoids, also affects how these molecules are attached into biological membranes. Although the way these molecules interact with reactive oxygen species differs due to their chain length and end groups, their in vivo behavior may differ from that seen in solution. Some of them, especially β-carotene and lycopene may also lose their antioxidant effects at high concentrations or high partial oxygen pressures [44,99,100].

## 6. Marine Carotenoids and Bioavailability

There are more than 40 carotenoids that can be obtained from diet and there are various health benefits of carotenoids to human health [11]. However, it is essential for every metabolite to reach sufficient concentration in target tissues to exert its bioactivity. Thus, bioavailability is a key concept for evaluating health benefits of carotenoids. In this concept, effects of digestive system, absorption and biotransformation should be observed for clear understanding.

Fucoxanthin and astaxanthin are the most abundant carotenoids from marine organisms consequently these two are the most studied marine carotenoids for their bioavailability. Fucoxanthin is the most ample carotenoid in the nature and it is mostly found in marine environment [101]. Because of the health benefits of fucoxanthin, there are various in vitro, in vivo and clinical studies for its bioavailability. Sugawara et al. had investigated bioavailability of Fucoxanthin and ten other carotenoids in an in vitro study via using Caco-2 cellular line, which is originated from gastrointestinal system. Results demonstrated that Fucoxanthin has one of the lowest bioavailability among other studied carotenoids [102]. In a further study same cell line was used and results revealed that, even though cells successfully absorb fucoxanthin, it is rapidly deacetylated and converted to Fucoxanthinol [103]. Correspondingly, another further in vitro study had showed that fucoxanthinol had transformed to amarouciaxanthin A by human hepatoma HepG2 cells [104]. Pharmacokinetic parameters of Fucoxanthin were also studied in vivo model, 0.105 mg of Fucoxanthin was administered to 6 mice via intragastric route [105]. Results showed that Fucoxanthin was not accumulating in the studied parts of mice such as lung, kidney, heart, erythrocytes, liver and spleen. On the opposite, metabolites of fucoxanthin, fucoxanthinol and amarouciaxanthin A reached their maximum concentration in 4 h and decreased steadily for 24 h. In the adipose tissue metabolites were still detectable for after a week. These results were corresponding with previous in vitro studies. Moreover, in a subsequent study, fucoxanthinol and amarouciaxanthin A accumulation in adipose tissue were confirmed after feeding mice with Fucoxanthin for 14 days [106]. It was theorized that with application of higher doses of fucoxanthin, it is possible to accumulate Fucoxanthin in mammalian tissues without any transformation. In a study with rats, it was shown that after 65 mg/kg intake of fucoxanthin, it reaches its maximum plasma level after 7.7 h with concentration of 29.1 µg/L [107]. Few studies were conducted on bioavailability of fucoxanthin in humans. In a study, 18 volunteers were orally administrated with Kambu extract which was containing 31 mg fucoxanthin and their blood samples were collected for 24 h. Results revealed that only fucoxanthinol was found in samples with maximum concentration of 44 nmol/L. Fucoxanthin and amarouciaxanthin A were not detected in plasma [108]. The outcome of the study declared by researchers was fucoxanthin in humans seems to have lower bioaccessibility in humans when compared to other carotenoids such as lutein and β-carotene. When considered the low bioavailability of fucoxanthin, different strategies were developed to increase it. In a study, rats were fed with brown seaweed, *Padina tetrastromatica* L., and chitosan-glycolipid hybrid nanogels were prepared for increasing the bioavailability. Results demonstrated that nanogels were improved the bioavailability more than 3-fold when compared to control group [109]. In addition, another study showed that fucoxanthin enriched *Phaeodactylum tricornutum* bioavailability can be increased with preparation of nanoparticles coated with casein and chitosan. C_max_ value was increased from 10.29 ± 0.1 to 33.66 ± 0.5 pmol/mL [110]. In a more recent study, it has been shown that digalactosylmonoacylglycerol and sulfoquinovosylmonoacylglycerol presence significantly increases both uptake and transport of fucoxanthin in Caco-2 cells [111].

There are numerous studies indicating that astaxanthin is highly valuable compound for human health [18]. This valuable potential of astaxanthin for human health leads to studies of its bioavailability. In a double blind randomized study, 28 health volunteers were ingested 250 g farmed or wild salmon which contain 5 µg Astaxanthin per gram for 2 weeks. The source of astaxanthin for wild salmon is krill while farmed ones were fed with synthetic type. Results showed that aqua cultured salmon leads to higher bioavailability; aquacultured salmon group reached 42 nmol/L while wild salmon group measured 27.3 nmol/L in day 3. It was reported that difference in bioavailability originated from isomerization [112]. In another study, pharmacokinetics of astaxanthin was examined in 3 middle-aged male volunteers with consumption of 100 mg racemic astaxanthin mixture in olive oil. Results demonstrated that astaxanthin concentration reached its maximum level after 7 h (1.3 mg/mL) and it was still observable in plasma for 72 h [113]. Results indicate that astaxanthin reaches significant levels in plasma without any biotransformation contrasting Fucoxanthin. Another clinical study was investigated the difference in bioavailability of astaxanthin between commercial food supplement and lipid-based formulation containing *Haematococcus pluvialis* green microalgae with 40 mg of astaxanthin [114]. Results revealed that lipid-based formulation achieved approximately 4-fold increment in bioavailability (1347 to 4960 µg h/L). In addition *C*_max_ value increased significantly from 55.2 to 191.5 µg/L. It is known that bioavailability may be affected various parameters such as sex, age, obesity, smoking, lifestyle, etc. In another study, effect of smoking on astaxanthin bioavailability and pharmacokinetic parameters was investigated on human volunteers. Results indicated that smoking significantly affected pharmacokinetic parameters for astaxanthin by reducing the half-life of elimination 30.5 h to 18.5 h. In addition, AUC value is lower in smoking group when compared to non-smoking volunteers, 6.52 and 7.53 µg h/L, respectively [115]. Even though, astaxanthin reaches sufficient plasma levels without biotransformation, still some metabolites of astaxanthin in human body were detected. 3-hydroxy-4-oxo-β-ionone, 3-hydroxy-4-oxo-β-ionone, 3-hydroxy-4-oxo-β-ionol, 3-hydroxy-4-oxo-7,8-dihydro-β-ionol was detected in two human volunteers via GC-MS analysis [116].

## 7. New Delivery Systems Used to Increase Marine Carotenoids Bioavailability

Encapsulation is one of the quality protection techniques of sensitive agents and is a method for production of them with new valuable properties. Recently, nanotechnology has contributed significantly to the development of drug carrier systems (biopolymeric and lipid-based carriers such as nanoliposomal formulations, surfactant-based nano-carriers, nanoemulsions, nano-structured lipid carriers) that can encapsulate compounds with poor stability, protect them from undesirable reactions, provide therapeutic effect at a low dose, improve targetability, and increase their activity [9,117]. In this regard, there are many studies on the application of encapsulated carotenoids in different nano-based drug delivery systems.

### 7.1. Application of Biopolymeric Nanocarriers for Encapsulation of Carotenoids

One of marine carotenoids, fucoxanthin has many effects, such as antioxidant, anti-obesity and anti-cancer effects [118] but it has high sensitivity when exposed to weather, thermal or light factors. Vo et al. evaluated to characterization and storage stability of encapsulated extracted fucoxanthin-rich oil (FO) from y using supercritical carbon dioxide with sunflower oil (SFO) and polyethylene glycol (PEG) as co-solvent and biodegradable coating at optimized conditions (mixing ratio of FO and PEG, temperature, and pressure). By using PEG as a biodegradable coating material, approximately 82% fucoxanthin encapsulation and a higher antioxidant activity of FO were found significantly than SFO and trolox [119].

Ravi et al. aimed to increase the stability and bioavailability of carotenoid such as fucoxanthin, encapsulated in chitosan, a biodegradable cationic polysaccharide, and glycolipid hybrid nanogels. Bioavailability studies were performed for investigate the effect of nanoencapsulation of fucoxanthin with glycolipid via micellization in vitro (simulated gastric and intestinal digestion). The bioavailability of fucoxanthin from chitosan-glycolipid hybrid nanogels was the highest compared to chitosan nanogels without glycolipid, mixture of fucoxanthin with glycolipid and control groups. The enhanced stability and bioavailability of fucoxanthin was observed by nanoencapsulation with chitosan and glycolipid [120].

As a continuation study, Ravi et al. prepared chitosan-glycolipid nanogels and investigated cellular uptake and anticancer efficacy of fucoxanthin on human colon cell line (Caco-2). They found that the low cell viability significantly at nanoencapsulated fucoxanthin with chitosan and glycolipid compared to without glycolipid for 48 h exposure. Chitosan-glycolipid hybrid nanogels successfully induced apoptosis in human colon cancer cells and suppressed ROS production by suppressing Bcl-2 protein associated increased levels of Bax through caspase-3 steps and suppressed cell viability [121].

Koo et al. developed casein nanoparticles (C-NP) and chitosan-coated nanoparticles (C-NP-CS) containing a valuable marine carotenoid, fucoxanthin which extracted from the microalga *Phaeodactylum tricornutum* for improvement of water solubility and its bioavailability. Nanoparticles characterized in terms of their size, morphology, structure, zeta potential, polydispersity index, encapsulation efficiency and adsorption to mucin. Increased retention and adsorption by mucin were observed with using cationic biopolymer chitosan coating and fucoxanthinol absorption to the blood circulation was found two-fold higher than with using C57BL/6 mice compared to C-NP [110].

Edelman et al. studied on new approach to improve the bioactivity of astaxanthin which is sensitive to oxidation and has very low bioavailability. Potato protein was approved by FDA and potato protein-based astaxanthin nanoparticles were designed because of be possible to reduce the dose of the required bioactive substance, to ensure the therapeutic effect and to increase solubility and oral bioavailability. Encapsulated astaxanthin was shown that ~80% simulated gastric and intestinal digestion and 11 times higher bioavailability was obtained compared to unencapsulated astaxanthin. Potato protein nanoparticles can be considered as a suitable drug delivery system to increase the bioavailability of hydrophobic compounds [122].

Application of biopolymeric nanocarriers for encapsulation of carotenoids is summarized in Table 1.

### 7.2. Application of Different Lipid-Based Nanocarriers for Encapsulation of Carotenoids

Many lipid based nanoformulations such as nanoliposomes, niosomes, solid lipid nanoparticles and nanostructured lipid carriers, were developed for improving biological activity, physicochemical stability, digestibility, antioxidant potential, resistibility and in vitro bioaccessibility.

Cordenonsi and co-workers aimed to develop nanostructured lipid carriers containing fucoxanthin for skin application to prevent skin hyperproliferative diseases. Hyperproliferation of the skin could be controlled and skin integrity could be restored with the presence of fucoxanthin. Nanostructured lipid carriers were coated with chitosan and biopharmaceutical properties (bio/mucoadhesion and wound healing) were improved by combining the advantages of chitosan. It stated that these nanostructured lipid carriers were promising approach with fucoxanthin to control skin hyperproliferation and maintain skin integrity in psoriatic skin [123].

Hama et al. evaluated the free radical scavenging effect of a useful antioxidant, astaxanthin loaded liposomes compared with the activity of the well-known antioxidants encapsulated β-carotene and α-tocopherol that stated that was the first report. The liposomal formulation provided encapsulation of high concentrations of astaxanthin and it was obtained that more potent hydroxyl radical scavenging activity significantly in aqueous solution was than either encapsulated β-carotene or α-tocopherol. Moreover, astaxanthin liposomes prevented cytotoxicity caused by hydroxyl radical in cultured NIH-3T3 cells [124].

Li et al. evaluated the protective effects of astaxanthin liposome (Asx-L) on photodamage by ultraviolet (UV)-B in mice skin. Topically treatment with 4 mL 0.2% astaxanthin or 4 mL 0.2% Asx-L were applied to groups (UVB light injury + astaxanthin and UVB light injury + Asx-L) 10 min before the irradiation for two weeks. The histological changes of skin, Ki-67, 8-hydroxy-2’-deoxyguanosine (8-OHdG), SOD activities and serum MMP-13 were measured. The pathological changes of skin tissues were significantly improved by topical Asx-L with decreased expressions of Ki-67, MMP-13 and 8-OHdG and increased SOD activity, due to strong antioxidation of Asx-L [125].

In the same study, Hama et al. designed Asx-L and evaluated prevention of UV-induced skin damage. Astaxanthin, which is accepted as a strong antioxidative carotenoid in biological membranes, is difficult to apply topically to the skin due to its poor water solubility. In this study, researchers tried to solve this problem by preparing Asx-L, which are suitable drug delivery systems for topical application to the skin. Asx-L has been shown to have strong cleaning ability against singlet oxygen production due to chemiluminescence in the water phase. When Asx-L was applied to the skin before UV exposure, skin thickening, and collagen reduction were prevented. The production of melanin was inhibited with topical application of Asx-L. As a result, application of a liposomal formulation containing Asx, it is possible to prevent skin damage caused by UV-induced [126].

Peng et al. prepared astaxanthin encapsulated in liposomes to eliminate such poor physicochemical properties of astaxanthin such as instability, low bioavailability and poor water solubility. Astaxanthin is a powerful oxygen radical scavenger and its efficacy on Hep3B and HepG2 cell lines was evaluated because of it was beneficial for carcinoma prognosis. Encapsulated astaxanthin showed apparently improved stability and permeability thus the total transport time is 7.55 h for encapsulates astaxanthin and 6.00 h for free astaxanthin. It was obtained that more antioxidant effect on intracellular antioxidant enzymes such as superoxide dismutase, catalase and glutathione S-transferase with astaxanthin liposomes compared to free astaxanthin and effectively facilitates apoptosis in the Hep3B cell line more than HepG2 cell line. The findings indicated that poor bioavailability of Asx can be improved and the prognosis of gamma radiation therapy with liposomal encapsulation [127].

In a previous study, Tamjidi et al. aimed to design nanolipid-based carriers (NLCs) containing astaxanthin which has instability, low bioavailability, and poor water solubility, to investigate the effect of contents of oil and surfactant on characteristics of astaxanthin-NLCs, and to optimize the characteristics and composition of formulation by response surface methodology. Tween 80 and lecithin and oleic acid, glyceryl behenate were selected as emulsifier and suitable lipids, respectively. The optimum formulation of Asx-NLCs with ideal properties was prepared with oleic acid Tween 80 and was different in terms of physical properties and storage stability. According to the data obtained in this study, NLCs could be used as a carrier system with potentially suitable physical stability for the administration of Asx and other lipophilic compounds into pharmaceutical products [128].

Barros et al. astaxanthin and peridinin, two typical carotenoids of marine microalgae included in phosphatidylcholine multilamellar liposomes and evaluated as inhibitor effects of lipid oxidation. Astaxanthin strongly decreased lipid damage when the lipoperoxidation promoters such as H_2_O_2_, tert-butyl hydroperoxide or ascorbate. Fe^+2^:EDTA were added simultaneously to the liposomes which were prepared with egg yolk phosphatidylcholine which offers suitable oxidation targets for ROS. To check for antioxidant activity of carotenoids which was also associated with their effect about membrane permeability, peroxidation processes started by adding promoters to encapsulated Fe^+2^ in the inner aqueous solution in liposomes. Consequently, astaxanthin was a more effective antioxidant (26%) at H_2_O_2_ and ascorbate-induced lipoperoxidation at Fe^+2^ liposomes. Peridine showed a more moderate inhibition of the lipid oxidation process (17.7%) due to it could have limited the permeation of the peroxidation agents [129].

In another study, effect of astaxanthin on the structural properties of liposome membranes was evaluated by Pan et al. They stated that liposomal encapsulation could be greatly increased water dispersibility of astaxanthin and also an effective way to constantly supply astaxanthin in the body [130].

Rodriguez-Ruiz et al. developed nanostructured lipid carriers to improve antioxidant activity of Asx using a green process with SFO as liquid lipid. Antioxidant activity of encapsulated astaxanthin was measured using the physicochemical lipophilic α-tocopherol equivalent antioxidant capacity (TEAC). It was demonstrated that nanostructured lipid-based carriers have suitable potential to maintain or stabilize the antioxidant ability of astaxanthin and could be excellent candidates as antioxidant delivery systems for cosmetics and nutraceuticals [131].

Bhatt et al. conducted to intranasal delivery of astaxanthin-loaded solid lipid nanoparticles (Asx- solid lipid nanoparticles (SLNs)) to improve brain targeting for neurological disorders. Double emulsion solvent displacement method was used for preparing SLNs. SLN formulation (50 mg stearic acid, 6.11% bioactive, and poloxamer 188, lecithin ratio of 1:6) was optimized with a 213 nm particle size. On the pheochromocytoma-12 cell line, the antioxidant potential of Asx-SLNs was evaluated against H2O2 induced toxicity. Radiolabeled nanoparticles were found to be 96–98% stable even for 48 h and higher drug concentration in the brain was achieved by intranasal administration, which was compared to 99mTc labeled nanoparticles intravenous route and confirmed by gamma scintigraphy analysis. It was shown that this nasal system can provide the most neuroprotection in neurological disorders from oxidative stress under in vitro conditions [132]. Shanmugapriya and colleagues designed to emulsion-based delivery systems to increase the bioavailability of astaxanthin and α-tocopherol as active compounds for various biomedical applications. Anticancer potential of astaxanthin-α-tocopherol nanoemulsion prepared with spontaneous and ultrasonication emulsification methods was evaluated with optimizing production conditions for better resistance and toxicity testing against microbial infections. It could be said that this delivery system may be a starting point for prospective studies for cancer treatment applications [133].

Rostamabadi et al. summarized some nano-based drug delivery systems in review. One of them was nanoliposomal formulations, consisting of lutein, different lipids and cholesterol as a stabilizer were developed, characterized and evaluated in terms of microstructure analysis using TEM, particle size, polydispersity, zeta potential, in vitro release and stability and cytotoxicity studies. These formulations provided to increased physicochemical stability, water dispersibility, therapeutic effects (antioxidant and anticancer) of lutein. In other studies with nano-emulsion based delivery system, formulation components, process steps, emulsifier types and concentrations storage pH and temperature played a key role on stability and effectiveness of lutein. Emulsion formulation revealed proper stability thus coating oil droplets in emulsions was increased the physicochemical stability of lutein compared to uncoated. In vitro bioaccessibility and chemical stability of lutein emulsions were found higher than non-capsulated forms. Protection of bioactive lutein against UV irradiation was evaluated and in depression lutein was found that 14%, 6–8%, and only 0.06% from nanoemulsion, NLCs and SLNs. With these systems, highly antioxidant effect of lutein was found with 85% encapsulation efficiency and 98% suppressing free radicals [117].

It was stated that nutraceutical colorant of the carotenoid family, β-carotene has poor bioavailability and low physicochemical stability. To achieve sustained release, to target and to increase the application of β-carotene as a bioactive, numerous studies reported in some lipid-based and nano-scale carriers, such as nanoliposomes, SLNs, NLCs, nanoemulsions, and niosomes. In generally, β-carotene was encapsulated successfully into these nanocarriers and stability and biological activity of β-carotene were increased depending on the formulation components, interactions of β-carotene with them and methods [117].

Unlike other nanocarriers, in β-carotene loaded niosomal formulation, good resistibility to light, elevated temperatures and oxidative stress created by free radicals were obtained. In culture medium, β-carotene maintained resistant after 4 days and it was easily taken up through cells at 0.1–2 µM concentrations [134].

Similarly, lycopene which provides beneficial effect on human health, has low stability on different conditions such as light, high temperatures, oxygen, chemical reactions and environmental factors. Some nanocarriers (nanoliposomes, SLNs, NLCs, nanoemulsions and niosomes) were developed to eliminate the destructive effects and to enhance stability and bioactivity of lycopene. In one study, lycopene loaded niosomes were prepared successfully to protect the lycopene activity and increased its bioavailability. Antiproliferative activity was evaluated through MCF-7 and HeLa cell lines and niosomal formulation was shown perfect response in a dose-dependent manner. Therefore, anticancer activity of potential of lycopene was confirmed [135]. In another niosomal formulation containing lycopene, antidiabetic effect was assessed and effectiveness of formulation was stated [136].

Application of lipid-based nanocarriers for encapsulation of carotenoids is summarized in Table 2.

## 8. Application of Emulsion-Based Systems for Encapsulation of Different Carotenoids

Shu et al. prepared oil in water (O/W) thermo-stable nanoemulsions containing astaxanthin and stabilized with ginseng saponins as a natural surfactant via a high-pressure homogenization method to investigate the effects of pH, ionic strength, heat treatment and different temperatures (5, 25, and 40 °C) on the stability of droplets and/or astaxanthin nanoemulsions. A good long-term stability was shown against droplet growth during 15 days of storage at various temperatures (5, 25, and 40 °C) and the nanoemulsions were stable without droplet coalescence against thermal treatment (30–90 °C, 30 min), Their finding shown that nanoemulsion-based system could be suitable for delivering oil-soluble bioactive compounds such as astaxanthin and these studies need to be continued [137].

Khalid et al. evaluated the effect of modified lecithin (ML) and sodium caseinate (SC) on the formulation, stability and in vitro digestive behavior and bioavailability of astaxanthin-O/W nanoemulsions using high-pressure homogenization method. It was shown that successful formulation of astaxanthin-loaded nanoemulsions and emulsifiers effectively stabilized the O/W nanoemulsions but much higher bioaccessibility of astaxanthin was observed in ML-stabilized nanoemulsions mainly due to greater formation of mixed micelles compared to SC-stabilized nanoemulsions [138].

Liu et al. studied on effect of three long chain triglyceride (LCT) oils and evaluated bioactivity of using encapsulated astaxanthin in O/W nanoemulsions with simulated gastrointestinal tract (GIT) model. Astaxanthin-loaded nanoemulsions with greater bioaccessibility compared to free nanoemulsions, and this indicated that mixed micelles produced by LCT digestion resulted in greater solubility of this lipophilic bioactive substance [139].

In another nanoemulsion study, Affandi et al. investigated the effect of different surfactants on the physicochemical characteristics and stability of astaxanthin-loaded nanoemulsions over 90 days. Droplet size/size distribution of the nanoemulsions mentioned above depended on homogenization pressure/cycles and the type/concentration of surfactants (lecithin or Tween 80). For effective nanodelivery system of astaxanthin, it was found that 2% *w*/*w* astaxanthin and 4% *w*/*w* surfactant at 9000 rpm prehomogenization speed (~5 min) as optimized conditions [140].

Shanmugapriya and colleagues designed to emulsion-based delivery systems to increase the bioavailability of astaxanthin and α-tocopherol as active compounds for various biomedical applications. Anticancer potential of astaxanthin-α-tocopherol nanoemulsion prepared with spontaneous and ultrasonication emulsification methods was evaluated with optimizing production conditions for better resistance and toxicity testing against microbial infections. It could be said that this delivery system may be a starting point for prospective studies for cancer treatment applications [133].

Tan et al. designed a novel delivery system as named chitosan coated liposomes (chitosomes) containing lutein which included in the carotenoid family, for improving stability [141]. In another study, lutein loaded particle system was prepared with using polyvinylpyrrolidone and Tween 80 as a stabilizer and emulsifier respectively by Zhao et al. They found that this particle system had convenient water dispersibility and enhanced stability compared to free lutein [142].

In other studies with nano-emulsion based delivery system, formulation components, process steps, emulsifier types and concentrations storage pH and temperature played a key role on stability and effectiveness of lutein. Emulsion formulation revealed proper stability thus coating oil droplets in emulsions was increased the physicochemical stability of lutein compared to uncoated. In vitro bioaccessibility and chemical stability of lutein emulsions were found higher than non-capsulated forms. Protection of bioactive lutein against UV irradiation was evaluated and lutein depression was found that 14%, 6–8%, and only 0.06% from nanoemulsion, NLCs and SLNs. With these systems, highly antioxidant effect of lutein was found with 85% encapsulation efficiency and 98% suppressing free radicals [117].

Application of emulsion-based systems for encapsulation of different carotenoids is summarized in Table 3.

## 9. Conclusions

There is a worldwide leaning on natural ingredients in food, nutraceutical, and cosmetics. It has been known that marine organisms serve as an extensive source for bioactive secondary metabolites due to their viability in extreme environments and conditions. In particular, carotenoids obtained from marine organisms displayed promising activity against various oxidative stress related disorders, and epidemiological studies revealed that the populations consuming carotenoid rich diets have lower oxidative stress related disorder risk. Although several activities of such compounds were shown by in vitro, in vivo, and several clinical studies, there is a need for advanced biological and molecular studies revealing the efficacy, activity mechanism, and metabolism. Bioavailability is an important factor to consider when developing suitable formulations with health benefits, since new delivery systems were produced for better bioavailability.

## Figures and Tables

**Table 1:** Application of biopolymeric nanocarriers for encapsulation of carotenoids.

*Nanocarriers*	*Carotenoids*	*Results and Benefits*	*References*
***Chitosan-glycolipid hybrid nanogels***	Fucoxanthin	The bioavailability of fucoxanthin from chitosan-glycolipid hybrid nanogels was the highest compared to chitosan nanogels without glycolipid, mixture of fucoxanthin with glycolipid and control groups. Enhanced stability and bioavailability by nanoencapsulation.	[120]
		The low cell viability significantly at nanoencapsulated fucoxanthin compared to without using glycolipid and induced apoptosis in Caco-2 cells and suppressed ROS production.	[121]
***PEG biodegradable coated nanoparticles***		Approximately 82% fucoxanthin encapsulation and a higher antioxidant activity significantly than sunflower oil and trolox.	[122]
***Casein nanoparticles and chitosan-coated nanoparticles***		Increased retention and adsorption and two-fold higher absorption to the blood circulation than non-coated nanoparticles.	[110]
***Potato protein-based polymeric nanoparticles***	Astaxanthin	~80% simulated gastric and intestinal digestion and 11 times higher bioavailability compared to unencapsulated astaxanthin.	[122]

**Table 2:** Application of lipid-based nanocarriers for encapsulation of carotenoids.

Nanocarriers	Carotenoids	Results and Benefits	References
***Nanostructured lipid carriers (NLCs)***	Fucoxanthin	NLCs were promising approach with fucoxanthin to control skin hyperproliferation and maintain skin integrity in psoriatic skin.	[123]
***Liposomes***	Astaxanthin	More potent hydroxyl radical scavenging activity of astaxanthin significantly than either encapsulated β-carotene or α-tocopherol and prevented cytotoxicity on NIH-3T3 cells.	[124]
		The pathological changes of skin tissues were significantly improved and decreased expressions of Ki-67, MMP-13 and 8-OHdG and increased SOD activity were found.	[125]
		Singlet oxygen production could cleaned strongly. The production of melanin was inhibited.	[126]
		Improved stability and permeability, more antioxidant effect on intracellular antioxidant enzymes and effectively facilitated apoptosis.	[127]
		The suitable characteristics and composition of formulation, with ideal properties and storage stability were determined.	[128]
***NLCs***		Liposomal encapsulation could be greatly increased water dispersibility of astaxanthin.	[130]
		Improved antioxidant activity of astaxanthin was provided with NLCs that could be excellent candidates for cosmetics and nutraceuticals.	[131]
***Liposomes***	Astaxanthin and peridinin	Astaxanthin strongly decreased lipid damage with a more effective antioxidant at H_2_O_2_ and ascorbate-induced lipoperoxidation at Fe^+2^ liposomes.	[129]
***Solid lipid nanoparticles (SLNs)***	Astaxanthin	Radiolabeled nanoparticles were found to be 96–98% stable even for 48 h and higher drug concentration in the brain was achieved by intranasal administration, which was compared to 99mTc labeled nanoparticles intravenous route.	[133]
***Liposomes***	Lutein	Increased physicochemical stability, water dispersibility, therapeutic effects (antioxidant and anticancer) of lutein liposomal formulations.	[117]
***Nanoemulsion*** ***NLCs*** ***SLNs***		Highly antioxidant effect was found with 85% encapsulation efficiency and 98% suppressing free radicals.	
***Liposomes*** ***NLCs*** ***SLNs***	β-carotene	Successfully encapsulation of β-carotene and improved physicochemical stability during storage and increased biological activity of β-carotene.	
***Niosomes***		Good resistibility to light, elevated temperatures and oxidative stress with β-carotene loaded niosomal formulation. β-carotene maintained resistant after 4 days.	[134]
***Niosomes***	Lycopene	Protecting the lycopene activity and increased bioavailability. Perfect response in a dose-dependent manner and confirmed anticancer activity with niosomal formulation.	[135]
		62% encapsulation efficiency as a reproducible and efficient technique could increase anti-diabetic property.	[136]

**Table 3:** Application of emulsion-based systems for encapsulation of different carotenoids.

*Nanocarriers*	*Carotenoids*	*Results and Benefits*	*References*
***Nanoemulsion***	Astaxanthin	A good long-term stability was shown against droplet growth during 15 days of storage at various temperatures and the nanoemulsions were stable without droplet coalescence against thermal treatment.	[137]
		Emulsifiers (modified lecithin (ML) and sodium caseinate (SC)) effectively stabilized the nanoemulsions and higher bioaccessibility was observed in ML-stabilized nanoemulsions.	[138]
		The greater bioaccessibility compared to free nanoemulsions and greater solubility.	[139]
		Optimum formulation components and conditions was selected that 2% w/w astaxanthin and 4% w/w surfactant at 9000 rpm prehomogenization speed (~5 min) for 90 days.	[140]
		Increased the bioavailability of astaxanthin.	[141]
	Lutein	Increased the physicochemical stability of lutein with coating and higher in vitro bioaccessibility and chemical stability with nanoemulsions.	[117]
***Phospholipid-Chitosan vesicles (chitosomes)***		Convenient water dispersibility and enhanced stability compared to free lutein.	[142]
	β-carotene Lutein Lycopene	Combination with chitosan with electrostatic attraction onto the membrane surface successfully Improved stability and controlled release of carotenoids by chitosomes.	[141]

## Abbreviations

^1^O_2_	Singlet oxygen
4-HNE	4-hydroxy-2-nonenal
5-Fu	5-Fluorouracil
6-OHDA	6-hydroxydopamine
8-OHdG	8-hydroxy-2’-deoxyguanosine
AAPH	2,2’-Azobis(2-amidinopropane) dihydrochloride
ABTS	2,2′-azino-bis(3-ethylbenzothiazoline-6-sulfonic acid
Akt	Protein kinase B
ARE	Antioxidant responsive elements
BHA	Butylated hydroxyanisole
Car-OO^•^	Carotene-peroxyl radical
CAT	Catalase
C-NP	Casein nanoparticles
C-NP-CS	Chitosan-coated nanoparticles
DMADP	Dimethyl allyl diphosphate
DPPH	2,2-diphenyl-1-picrylhydrazyl
EDTA	Ethylene diamine tetra acetic acid
ERK1/2	Extracellular-regulated kinase ½
GCS	γ-glutamylcysteine synthetase
GPx	Glutathione peroxidase
GSH	Glutathione
GSTP1	Glutathione S-transferase
H_2_O_2_	Hydrogen peroxide
HIF-1α	Hypoxia inducible factor-1α
HOBr	Hypobromous acid
HOCl	Hypochlorous acid
Huh7	Human hepatic cellular carcinoma
IDP	Isopentenyl diphosphate
LDL	Low-density lipoprotein
LS-180	Human colorectal cancer cell line
MDA	Malondialdehyde
ML	Modified lecithin
MMP	Matrix metalloproteinase
MMPs	Matrix metallopeptidases
NADPH	Nicotinamide adenine dinucleotide phosphate
NF-κB	Nuclear Factor kappa B
NLCs	Nanolipid-based carriers
NOX	NAPDH oxidase
Nrf2−Keap1	Nuclear factor E_2_-related factor 2
O2	Molecular oxygen
O_2_^∙∙^	Oxygen radical
O_2_^⋅−^	Superoxide
O_3_	Ozone
OH^∙^	Hydroxyl
PI3K/Akt	Phosphatidylinositol-3 kinase
PTEN	Phosphatase and tensin homolog
RAR	Retinoid acid receptor
RNS	Reactive nitrogen species
RO^∙^	Alkoxy radical
ROO^.^	Peroxyl radical
ROS	Reactive oxygen species
RTK	Receptor tyrosine kinase
RXR	Retinoid X receptor
SC	Sodium caseinate
SH-SY5Y	Human neuroblastoma cell line
SLNs	Solid lipid nanoparticles
SOD	Superoxide dismutase
TAC	Plasma total antioxidant capacity
TBARS	Thiobarbituric acid reactive substances
tBuOOH	Tert-butyl hydro peroxide
TEAC	α-tocopherol equivalent antioxidant capacity
Trolox	8-tetramethylchroman-2-carboxylic acid
UV	Ultraviolet
VEGF	Vascular endothelial growth factor
